# Nesting behavior is associated with body weight and grip strength loss in mice suffering from experimental arthritis

**DOI:** 10.1038/s41598-023-49720-y

**Published:** 2023-12-28

**Authors:** Tamara Dietrich, Annette Aigner, Alexander Hildebrandt, Jérôme Weber, Mara Meyer Günderoth, Katharina Hohlbaum, Johannes Keller, Serafeim Tsitsilonis, Tazio Maleitzke

**Affiliations:** 1 Center for Musculoskeletal Surgery, Charité – Universitätsmedizin Berlin, Corporate Member of Freie Universität Berlin and Humboldt-Universität Zu Berlin, Berlin, Germany; 2 Julius Wolff Institute, Berlin Institute of Health at Charité – Universitätsmedizin Berlin, Berlin, Germany; 3 Institute of Biometry and Clinical Epidemiology, Charité – Universitätsmedizin Berlin, Corporate Member of Freie Universität Berlin and Humboldt-Universität Zu Berlin, Berlin, Germany; 4 German Centre for the Protection of Laboratory Animals (Bf3R), German Federal Institute for Risk Assessment (BfR), Berlin, Germany; 5 Department of Trauma and Orthopedic Surgery, University Medical Center Hamburg-Eppendorf, Hamburg, Germany; 6 BIH Charité Clinician Scientist Program, BIH Biomedical Innovation Academy, Berlin Institute of Health at Charité – Universitätsmedizin Berlin, Berlin, Germany; 7 Department of Orthopaedic Surgery, Copenhagen University Hospital - Amager and Hvidovre, Hvidovre, Denmark; 8 Department of Clinical Medicine, University of Copenhagen, Copenhagen, Denmark

**Keywords:** Experimental models of disease, Animal behaviour, Rheumatic diseases, Preclinical research

## Abstract

Objective animal health evaluation is essential to determine welfare and discomfort in preclinical in vivo research. Body condition scores, body weight, and grimace scales are commonly used to evaluate well-being in murine rheumatoid arthritis (RA) and osteoarthritis experiments. However, nest-building, a natural behavior in mice, has not yet been evaluated in wild type (WT) or genetically modified rodents suffering from collagen antibody-induced arthritis (CAIA). To address this, we analyzed nesting behavior in WT mice, calcitonin gene-related peptide alpha-deficient (αCGRP^-/-^) mice, and calcitonin receptor-deficient (Calcr^-/-^) mice suffering from experimental RA compared to healthy control (CTRL) groups of the same genotypes. CAIA was induced in 10–12-week-old male mice, and clinical parameters (body weight, grip strength, clinical arthritis score, ankle size) as well as nesting behavior were assessed over 10 or 48 days. A slight positive association between the nest score and body weight and grip strength was found for animals suffering from CAIA. For the clinical arthritis score and ankle size, no significant associations were observed. Mixed model analyses confirmed these associations. This study demonstrates that clinical effects of RA, such as loss of body weight and grip strength, might negatively affect nesting behavior in mice. Assessing nesting behavior in mice with arthritis could be an additional, non-invasive and thus valuable health parameter in future experiments to monitor welfare and discomfort in mice. During severe disease stages, pre-formed nest-building material may be provided to animals suffering from arthritis.

## Introduction

Protecting animal welfare in pre-clinical research is ethically crucial, but also directly impacts research results^1,2^ and is thus relevant for robust data acquisition and repeatability^3^.

The European directive 2010/63/EU provides guidelines for the assessment of animal welfare in preclinical research^4,5^. Standard assessments of general health in experimental mice include body weight and exterior appearance, including changes of skin, eyes, and fur care^5–7^.

For research models evaluating acute pain, the grimace scale is commonly used in laboratory animals^8^, yet in states of chronic pain it lacks accuracy^9,10^. Here, natural nesting behavior, which can be observed in wildlife and laboratory rodents^11,12^, may be used to obtain additional information on animal well-being. It was previously shown to be negatively correlated with post-operative pain, stress^13–15^, and neurological impairment^16^.

Rheumatoid arthritis (RA) is a chronic autoimmune disease, affecting symmetrical joints and extraarticular organs^17^. Articular degeneration is caused by multidirectional and pro-inflammatory signaling pathways, which affect the synovium, cartilage, and bone^18^, and subsequently impair joint movement.

We previously showed that peptides of the *Calca* family have distinct effects on the joint environment in murine collagen antibody-induced arthritis (CAIA). While the vasoactive calcitonin gene-related peptide alpha (αCGRP) acted pro-inflammatory and bone-protective^19^, the endogenous calcitonin receptor (CTR) exhibited an anti-inflammatory and bone-protective function^20^. Arthritis induction in preclinical murine models is accompanied by a temporary loss of body weight and grip strength^19,20^, yet nesting behavior has thus far not been evaluated in animals suffering from inflammatory joint diseases.

As part of an ongoing 3R investigation, nesting behavior in mice suffering from experimental RA and control (CTRL) animals from two previously conducted studies^19,20^ were monitored over either 10 or 48 days. We observed that mice deficient for αCGRP (αCGRP^-/-^) showed lower clinical arthritis scores and were able to preserve grip strength^19^, whereas mice deficient for the CTR (Calcr^-/-^) showed a clinical disease score, similar to that of arthritic wild type (WT) mice^20^. In this study, associations between nesting behavior and body weight, grip strength, clinical arthritis score, and ankle size were assessed over time.

We investigated whether nesting behavior could serve as an additional, non-invasive animal welfare surrogate marker, which may be used to identify disease stages where mice require additional assistance with nest-building due to impaired mobility.

## Results

### Comparisons between CAIA and CTRL mice

**Figure 1:**
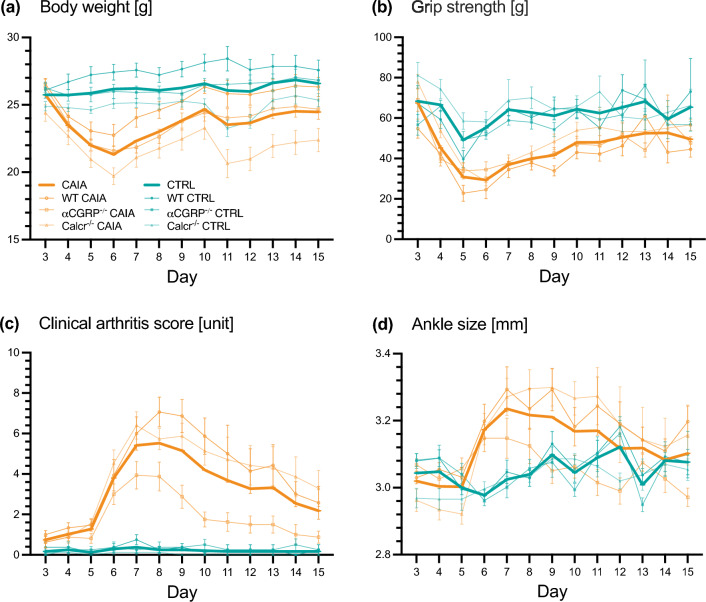
Clinical course of CAIA and CTRL animals. Longitudinal development of (**a**) body weight, (**b**) grip strength, (**c**) semi-quantitative clinical arthritis score, and (**d**) ankle size for CAIA and CTRL animals (WT, αCGRP^-/-^, Calcr^-/-^) from day 315 following arthritis induction. Displayed are mean values and standard error of the mean (SEM).

CTRL mice showed stable body weight gain during the primary observational period (inflammation phase, day 3–15). Contrarily, CAIA animals lost body weight following disease induction and had therefore a significantly lower body weight than CTRL animals overall (on average 3.59 g less [95% confidence interval (CI): -4.30; -2.87]). From day 6 onwards all CAIA mice started to regain weight (Fig. 1a, Table 1).

**Table 1:** Descriptive statistics and mixed-effect regression models for CAIA vs CTRL animals for nest score and clinical parameters based on all mouse-day observations between day 315.

Parameter		CAIA (n = 482)	CTRL (n = 252)	Effect estimate CAIA versus CTRL (95% CI)
Nest score (≥ 3) [unit]	n (%)	230 (59.6%)	187 (78.9%)	OR^1^ = 1.02 (0.45, 2.32)
	n missing	96	15	
Nest score (ordinal) [unit]	Median (IQR)	3.00 (1.25, 4.00)	4.00 (3.00, 5.00)	OR^2^ = 0.82 (0.39, 1.72)
	n missing	96	15	
Body weight [g]	Median (IQR)	23.50 (21.52, 25.50)	26.05 (25.00, 27.10)	Regression coefficient^3^ = -3.59 (-4.30, -2.87)
	n missing	0	0	
Grip strength [g]	Median IQR	42.83 (31.71, 53.52)	61.50 (51.71, 69.41)	Regression coefficient^3^ = -22.63 (-22.67, -17.59)
	missing	2	0	
Clinical arthritis score [unit]	Median IQR	2.00 (1.00, 5.00)	0.00 (0.00, 0.00)	Rate ratio^4^ = 17.35 (11.80, 25.50)
	missing	1	0	
Ankle size [mm]	Median IQR	3.10 (2.98; 3.24)	3.04 (2.98; 3.10)	Regression coefficient^3^ = 0.13 (0.08; 0.18)
	missing	1	0	

IQR = interquartile range; CI = confidence interval; n = number of observations; OR = odds ratio

^1^ Derived from mixed logistic regression, using a nest score ≥ 3 as the outcome.

^2^ Derived from mixed ordinal logistic regression.

^3^ Derived from mixed linear regression.

^4^ Derived from mixed negative binomial regression.

We observed a decrease in mean grip strength between day 3 and 5 for CAIA mice followed by a subsequent slow recovery. CTRL animals also showed a decline in mean grip strength, although they recovered faster. This habituation effect (indicated by a decline in grip strength) was previously described in healthy/CTRL animals^21,22^. Overall, CAIA mice showed significantly lower grip strength than CTRL animals (on average 22.63 g lower [-22.67; -17.59]) (Fig. 1b, Table 1).

The CTRL group showed no relevant clinical signs of arthritis. In comparison, CAIA mice received significantly higher clinical arthritis scores (overall a 17.35-fold higher rate [11.80; 25.50]). The inflammation phase of antibody-mediated arthritis peaked on day 8 followed by a transition into the resolution phase with a subsequent decrease of clinical signs of arthritis (Fig. 1c, Table 1).

Accordingly, the mean ankle size of CAIA animals started increasing between day 5 and 6 and reached its peak between day 7 and 8 (on average 0.13 mm bigger [0.08; 0.18]). CTRL animals fluctuated on a lower level, which increased over time (with increased age, body weight, and animal size) (Fig. 1d, Table 1).

**Figure 2:**
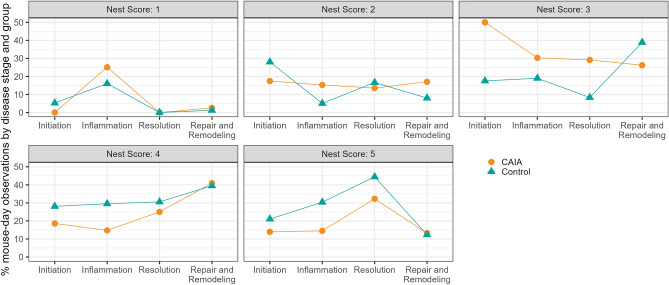
Relative distribution of nest scores (1–5) for CAIA and CTRL animals, based on observations per mouse and day for different disease stages of experimental RA.

Overall, CTRL mice attained a perfect nest score of 5 more frequently, except during the repair and remodeling phase (day 22–48). A low nest score of 1 was rarely observed, yet more frequently in CAIA animals during the inflammation phase (day 3–15) (Fig. 2). Specifically, during the inflammation phase (day 3–15), the odds of constructing a better nest (nest score ≥ 3) were not relevantly different between CAIA and CTRL animals (odds ratio (OR) = 1.02 [0.45; 2.32]). The results of the mixed ordinal logistic regression indicated slightly lower chances for CAIA animals to construct a better nest, but with a large uncertainty (OR = 0.82 [0.39; 1.72]; Fig. 2, Table 1).

Additional analyses conducted for the resolution and repair and remodeling phases suggest a convergence of values between CAIA and CTRL animals, as expected from the transient nature of the disease model (Supplementary Table S1 and Table S2 online).

### Associations between clinical parameters and nest score

To assess whether clinical parameters correlate with nesting behavior, we calculated repeated-measure Spearman correlations coefficients.

**Figure 3:**
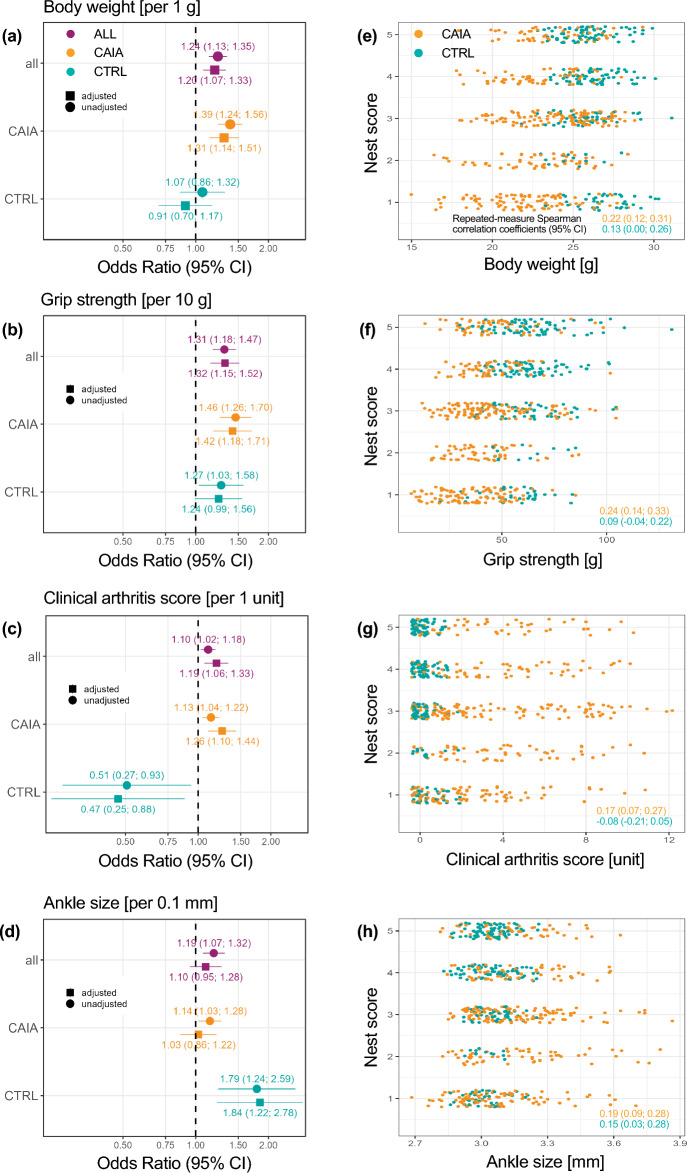
OR estimates derived from mixed logistic regression models, along with respective scatterplots, including repeated-measure Spearman correlation coefficients. Forest plots show unadjusted and adjusted OR estimates (along with 95% CI) derived from mixed logistic regression models for the dependent variable nest score ≥ 3 and independent variables (**a**) body weight [per 1 g], (**b**) grip strength [per 10 g], (**c**) clinical arthritis score [per 1 unit] and (**d**) ankle size [per 0.1 mm]; separately for all mice, CAIA, and CTRL animals. Scatterplots include repeated-measure Spearman correlation coefficients (along with 95% CI) for mouse-day observations for (**e**) body weight and nest score, (**f**) grip strength and nest score, (**g**) clinical arthritis score and nest score, and (**h**) ankle size and nest score; separately for CAIA and CTRL mice.

During the inflammation phase (day 3–15), body weight and nest score were fairly positively associated in CAIA mice (r = 0.22; [95% CI: 0.12; 0.31]), while the association for CTRL animals was lower (r = 0.13; [0.00; 0.26]) (Fig. 3e). Grip strength and nest score were also fairly positively associated in CAIA animals (r = 0.24; [0.14; 0.33]), and again a slight positive association was observed for CTRL mice (r = 0.09; [-0.04; 0.22]; Fig. 3f). The clinical arthritis score was slightly positively associated with the nest score in CAIA mice (r = 0.17; [0.07; 0.27]), with even a small negative association for CTRL animals (r = -0.08; [-0.21; 0.05]) (Fig. 3g). The nest score was also slightly positively associated with ankle size in CAIA (r = 0.19; [0.09; 0.28]) and CTRL mice (r = 0.15; [0.03; 0.28]) (Fig. 3h).

We fitted logistic mixed models with the dependent variable of a nest score ≥ 3, to evaluate associations between clinical parameters and nesting behavior. Among all mice, higher body weight, grip strength, clinical arthritis score, and ankle size in tendency increased the chances for a high-quality nest (Fig. 3a–d). Similar associations were found for CAIA mice only. An increase in body weight by 1 g increased the chances for a nest score ≥ 3 by 31% (adjusted OR = 1.31; [95% CI: 1.14; 1.51]), a 10 g increase in grip strength by 42% (adjusted OR = 1.42; [1.18; 1.71]), a 1 unit increase in arthritis score by 26% (adjusted OR = 1.26; [1.10; 1.44]). Increased ankle size, however, did not substantially alter the chances for a better nest (adjusted OR = 1.03; [0.86; 1.22]) (Fig. 3a–d).

For CTRL animals, on the other hand, the association between body weight and nest score was rather uncertain, with a tendency of increasing body weight to decrease the chances for a nest score ≥ 3 (adjusted OR = 0.91; [0.70; 1.17]). For grip strength, on the other hand, a 10 g increase increased the chances by 24% (adjusted OR = 1.24; [0.99; 1.56]). Interestingly, the association between clinical arthritis score and nesting was reversed. A 1 unit increase in the clinical arthritis score reduced the chances for a nest score ≥ 3 by 53% (adjusted OR = 0.47; [0.25; 0.88]). Last, an increase in ankle size by 0.1 mm increased the chances of a better nest by 84% (adjusted OR = 1.84; [1.22; 2.78]) (Fig. 3a–d).

The performed sensitivity analyses using an ordinal logistic regression showed similar results (Supplementary Table S3 online). Of note, CTRL animals generally received low clinical arthritis scores, as they did not suffer from experimental arthritis.

## Discussion

In this study, we demonstrated positive associations between body weight and grip strength and nesting behavior in group-housed mice suffering from experimental arthritis during the inflammation phase of CAIA. Evaluating nest-building is an easy-to-perform and non-invasive method to monitor animal health and can serve as an additional assessment of general health in chronic inflammatory disease models of group-housed animals, including autoantibody-mediated arthritis.

This 3R study was part of two previous experiments analyzing cartilage and bone quality in animals deficient for αCGRP and CTR during experimental RA, where arthritic αCGRP^-/-^ animals showed reduced^19^ and Calcr^-/-^ animals increased^20^ signs of inflammation during CAIA.

Each cage was assigned one genotype, and allocated animals were either treated according to the CAIA or the CTRL protocol. Our experimental setup allowed us to group animals in accordance with 3R principles, avoiding individual housing, and to still be able to retrieve information on nesting behavior for different genotypes and treatments^4^. To keep social structures stable and reduce distress, animals lived as littermates in groups of up to 10 from no later than 21 days of age^23^. With regard to territorial behavior of male mice, a recurring debate questions whether single housing is more appropriate than group-housing^24–28^. Several studies indicate that individual housing in mice leads to immunodeficiency, reduced coping with increased stress, and a higher incidence of morbidity^24,29,30^. Van Loo et al. further showed that male mice prefer group-housing over single-housing, indicated by increased appetitive behavior, independent of aggression and hierarchy^31^.

In contrast, we were previously able to show no differences in burrowing performance, social interaction, anxiety, and stress hormone concentrations in single-housed male mice, yet pair-housed mice built more complex nests^27^, which was taken into consideration in our current experimental design. In female mice we could show that pair-housing increased nesting and burrowing behavior, yet locomotor activity decreased^28^.

This study represents the first attempt to investigate associations between nesting behavior and clinical parameters commonly assessed in experimental RA, including body weight, grip strength, clinical arthritis score, and ankle size. Previous studies explored the well-being of animals suffering from experimental RA mainly through clinical and pain scores^32–34^. A more recent study suggested to survey spontaneous motor activity and preferred temperature and alterations in voluntary behavior as indicators of well-being in animals suffering from CAIA^35^. While assessing these parameters requires additional equipment and time, and potentially exposes animals to stress and additional pain, the evaluation of nesting behavior is quick and requires no additional technology. This may be one of the reasons why it is widely used to assess animal welfare in preclinical models of epilepsy^36^, Alzheimer’s disease^37^, and in post-surgical care^15,38^.

The use of nest scores seems especially appropriate as an indicator of compromised health in animal models which otherwise may not clearly indicate disease severity and pain. It may serve as an additional marker to help decide when to support animals with food, water, analgesics, and support in nest-building (provide pre-formed nests).

Our results showed that nesting behavior was positively associated with body weight and grip strength loss, but less so with clinical arthritis score or ankle size. This aligns with other studies suggesting that nest-building behavior is influenced more by the overall health status of animals rather than disease severity. In a study by Kumstel et al. mice suffering from pancreatic cancer showed no significant deviations in their nest-building behavior^38^. A more recent study demonstrated that nesting behavior in mice may also change depending on the type of intervention. For example, mice displayed reduced nest-building behavior after isoflurane anesthesia, but not after saline-i.p. injection^47^. In addition, a study on experimental osteoarthritis showed that mice suffering from surgically induced instability of the knee joint quickly returned to normal activity, including normal nesting behavior^48^. This supports our findings that nest-building behavior is not necessarily affected by disease severity but more by the clinical effects of RA, i.e. loss of body weight and grip strength.

We found weak positive associations for the CAIA group between nest score and body weight, and grip strength. Furthermore, we observed a weak positive association for nest score and clinical arthritis score in CAIA mice and inconclusive results for ankle size. The ambivalent associations of nest score and clinical arthritis score and ankle size could be explained by group-housing of animals that were differently affected by experimental arthritis (although similar in genotype and treatment protocol)^39,40^.

Due to our 3R-focused experimental design, this work has some limitations. First, we attributed one nest score to all animals from the same cage, which may over- or underestimate the impact of the disease on individual nest-building capacities. Mice with milder RA symptoms may therefore have contributed more to nest-building than more severely affected animals.

To assess individual nest-building contributions, subcutaneous implantation of radio-frequency identification (RFID)-tracers allows real-time tracking of each mouse’s position within the cage. Alternatively, color-coding also allows tracking mice via a camera system. However, tracer implantation is invasive and exposes animals to surgical and post-surgery stress and pain^41–44^. Importantly, this method lacks detailed information on specific behavioral traits associated with the tracked position^44^, making it challenging to discern whether a mouse is actively contributing to nest-building or merely resting within the nest. Color-coding on the other hand is prone to tracking errors based on available reports^44–46^. The most effective approach for comprehensive tracking of position and activity for each mouse is RFID-video assisted tracking, which is expensive and requires additional equipment, time, and personnel^44^. Considering the drawbacks associated with these elaborate technical tools, this study aimed to assess nesting as an affordable and easy-to-assess health parameter in mice suffering from experimental RA.

Second, in contrast to most CAIA studies, our mice received the analgesic metamizole via drinking water throughout the experiment. Metamizole is considered to have good analgesic but only very weak anti-inflammatory effects^49^. In comparison to studies performed without analgesics, the clinical expression of experimental RA in our study was similar to previously published data^50,51^. Analgesia could however be another factor affecting nesting behavior of animals, yet all mice received metamizole at the same dose, which reduces the probability of relevant interindividual differences.

Nevertheless, this study was the first to systemically investigate impaired nesting behavior in mice affected by the clinical effects of RA, such as loss of body weight and grip strength.

## Conclusion

The evaluation of nesting behavior in mice affected by arthritis may serve as an additional health parameter worth considering in future murine experiments.

We conclude that within the context of chronic inflammatory joint diseases, relying exclusively on the nest score in group-housed mice is not recommended. However, it may prove valuable as a supplementary marker for identifying cages with animals potentially requiring intensified care.

While acknowledging the limitations associated with group-housing, our results shed light on the multifaceted nature of nest-building behavior in the context of experimental RA. Our findings contribute to a broader understanding of the factors influencing this behavior and underline the need for further research to unravel the intricate relationships between individual health, group dynamics, and nesting in laboratory mice.

## Methods

### Animals, experimental design, and housing

This 3R study was part of two previous experiments^19,20^ where CAIA was induced by an intraperitoneal (i.p.) injection of 8 mg (0.4 mL) of ArthritoMab arthritis-inducing antibody cocktail (20 mg/mL) in 15 WT, 16 αCGRP^-/-^, and 16 Calcr^-/-^ mice on day 0, followed by an arthritis-boosting i.p. injection of 100 μg (0.2 mL) of lipopolysaccharide (LPS) (0.5 mg/mL) (both MD Bioproducts, Oakdale, MN, USA) on day 3. Accordingly, WT CTRL (n = 8), αCGRP^-/-^ CTRL (n = 8) and Calcr^-/-^ CTRL mice (n = 8) received i.p. injections of sterile phosphate-buffered saline (PBS) on day 0 and day 3. All animals were male, 10–12 weeks old, backcrossed at least seven times into a pure C57Bl/6 J genetic background, kept at a 12 h light/12 h dark cycle, group-housed, with access to water and standard diet (Rat/Mouse–Maintenance, ssniff, Soest, Germany) ad libitum. Analgesic metamizole was continuously administered via the drinking water from day 0 onwards (1350 mg/kg body weight/day). Dry food was provided on the cage floor during acute arthritis, and clinically severely affected mice received 0.01 ml (5 mg) metamizole subcutaneously.

Acute and chronic effects of CAIA were assessed either 10 or 48 days after induction. Humane endpoints and subsequent premature animal sacrification included weight loss of > 30% compared to baseline without recovery within 24 h, limping, as well as avoidance of movement and grooming. In line with 3R principles, individual housing was avoided^4^.

All animals were kept in different group sizes in type III cages (measurement: 42.5 × 27.6 cm) (Zoonlab, Castrop-Rauxel, Germany) and monitored for 10 or 48 days (Table 2). Naturalistic nest score^9^, body weight, grip strength, clinical arthritis score^19^, and ankle size were assessed daily by the same investigator (A.H.) between 9 am and 12 pm. Animal handling included cupping and tail handling. Group sizes and animal distribution are shown in Table 2 and sample size calculations were previously described^19,20^. Humane endpoints were reached prior to their regular study termination in 3 WT CAIA, 2 αCGRP^-/-^ CAIA, and 1 Calcr^-/-^ CAIA mice.

**Table 2:** Distribution of mice per cage divided by genotypes.

Genotype	Cage	Number of animals until day 10	Number of animals from day 11 onward
WT	CTRL Cage 1	8	4
	CAIA Cage 1	10	2
αCGRP^-/-^	CTRL Cage 1	2	2
	CTRL Cage 2	2	2
	CTRL Cage 3	3	0
	CAIA Cage 1	1	0
	CAIA Cage 2	5	5
	CAIA Cage 3	3	0
	CAIA Cage 4	2	0
Calcr^-/-^	CTRL Cage 1	4	0
	CTRL Cage 2	4	4
	CAIA Cage 1	2	0
	CAIA Cage 2	3	3
	CAIA Cage 3	6	0
	CAIA Cage 4	5	4

As individual housing was avoided, only a limited number of cages could be attributed to one genotype or treatment (Table 2), which is why genotype comparisons were omitted. Therefore, individual cages were assigned either to CTRL or CAIA groups.

Overall, 8 WT CTRL, 10 WT CAIA, 7 αCGRP^-/-^ CTRL, 14 αCGRP^-/-^ CAIA, 8 Calcr^-/-^ CTRL, and 16 Calcr^-/-^ CAIA (= 40 CAIA and 23 CTRL mice) were analyzed.

To quantify results, special nesting material was used. Two autoclaved nestlets (compact crinklet natural) and three bedding rolls (both SAFE®, Rosenberg, Germany) were provided in each cage once a week (Supplementary Figure S1 online). Cages were further equipped with a plastic house and tube (both Zoonlab, Castrop-Rauxel, Germany) and special fine bedding from SAFE® (SAFE®, Rosenberg, Germany) with a filling height of 1–1.5 cm. Cages were cleaned once a week.

### Arthritis assessment

Each mouse was weighed once daily, followed by arthritis assessment based on the semi-quantitative clinical arthritis score^50^. A score of 0–3 was given according to redness, swelling, and number of affected digits for each limb. Thus, one animal could reach a maximum score of 12. Ankle size was measured using a digital caliper (I Gaging, Granada Hills, California, United States) to assess the maximum medial to lateral width, and mean values of both back paws were calculated. Grip strength of front paws was assessed using the BIO-GS3 grip strength test (Bioseb, Vitrolles, France). The maximum pulling force was measured 5 consecutive times and mean values were calculated. These results have been published previously^19,20^.

### Naturalistic nest score

**Figure 4:**
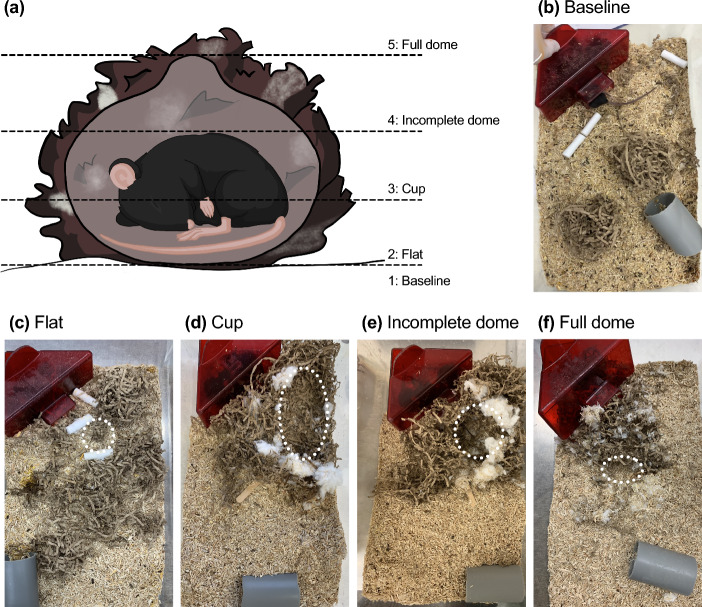
Schematic and real-life representation of naturalistic nest score. (**a**) Schematic and (**b**)-(**f**) real-life images of the naturalistic nest score based on Gaskill et al.^9^. White dotted circles indicate the nest center, except in (**f**), where the entrance to the nest is shown.

Nesting behavior was evaluated during 9 am and 12 pm using the nest score by Gaskill et al^9^. A score from 1–5 was attributed to each cage based on nest quality (Fig. 4a). Therefore, all mice held in the same cage received the same score, which was taken daily for either 10 or 48 days. If there was no visible interaction with the nest material, a nest score of 1 was assigned. If interaction with the nest-building material was observed, but no nest was built, a nest score of 2 was given. With a nest score of 3, a shallow nest was visible. A nest score of 4 indicated a nest which was clearly visible and towered over the mice. A nest score of 5 describes a nest that was completely enclosed and thus not visible from above (Fig. 4b–f).

Antibody-mediated arthritis can be divided into three main phases^52–54^: Inflammation (day 3–15), resolution (day 16–21), and repair and remodeling (from day 22 onward). If not stated otherwise, all reported results refer to the inflammation phase (day 3–15), being the clinically most recognizable phase of antibody-mediated arthritis^19,20,53^. In all analyses, daily nest scores were treated as individual observations for each mouse, assuming that the nest score did not vary within a cage. Overall, 181 mouse-day observations were collected during the inflammation phase (day 3–15).

### Statistical analysis

To estimate differences between CAIA and CTRL we used different mixed models with random intercept per cage, depending on the outcome variables nest score, body weight, grip strength, clinical arthritis score, and ankle size.

Repeated-measure Spearman correlation coefficients were calculated to assess correlations between nest score and body weight, grip strength, clinical arthritis score, and ankle size^55^. To address if nesting behavior was associated with clinical parameters of experimental RA, we applied mixed models with random intercept per cage, deriving unadjusted and adjusted OR estimates and 95% CI. Applying logistic regression, we employed a nest score of at least 3 as the dependent variable, indicating a sufficiently built nest. In the sensitivity analyses we applied an ordinal logistic regression with the full nest score as the dependent variable. Independent variables included body weight, grip strength, clinical arthritis score, and ankle size. Data analyses were conducted using R (version 4.1.1)^56^ and R packages^57–60^. Figures were created using R (version 4.1.1) and Prism V.9.5.0 (GraphPad, California USA).

### Ethical approval

Ethical approval (G-0044/18) was obtained from the local animal welfare organization (Landesamt für Gesundheit und Soziales in Berlin, Germany) and all experiments were carried out in accordance with the Directive 2010/63/EU for the protection of animals used for scientific purposes, the German Animal Welfare Act, and institution guidelines^61^. For data reporting and storage, we followed the internationally established ARRIVE guidelines^62^.

### Supplementary Information

Supplementary Information.

## Data Availability

The datasets used and/or analyzed during the current study are available in this published article and its supplementary information files and from the corresponding author on request.

## Abbreviations

αCGRP: Calcitonin gene-related peptide alpha
αCGRP^-/-^: Deficient for αCGRP
CAIA: Collagen antibody-induced arthritis
Calcr^-/-^: Deficient for calcitonin receptor
CI: Confidence interval
CTR: Calcitonin receptor
CTRL: Control
LPS: Lipopolysaccharide
OR: Odds ratio
PBS: Phosphate-buffered saline
RA: Rheumatoid arthritis
RFID: Radio-frequency identification
SEM: Standard error of the mean
WT: Wild type
