# Supplementary material for: Nesting behavior is associated with body weight and grip strength loss in mice suffering from experimental arthritis
Source: Sci Rep. 2023 Dec 28;13:23087. doi: 10.1038/s41598-023-49720-y (PMC10754866; doi:10.1038/s41598-023-49720-y)
Supplement: Supplementary file 1 — Supplementary Information. [file 41598_2023_49720_MOESM1_ESM.pdf]

Nesting Behavior is Associated with Body Weight and Grip Strength Loss in Mice Suffering from Experimental Arthritis

Tamara Dietrich [1,2], Annette Aigner [3], Alexander Hildebrandt [1,2], Jérôme Weber [1,2], Mara Meyer Günderoth [1,2], Katharina Hohlbaum [4], Johannes Keller [5], Serafeim Tsitsilonis [1,2], and Tazio Maleitzke [1,2,6,7]

1 Charité – Universitätsmedizin Berlin, corporate member of Freie Universität Berlin and Humboldt-Universität zu Berlin, Center for Musculoskeletal Surgery, Berlin, Germany

2 Berlin Institute of Health at Charité – Universitätsmedizin Berlin, Julius Wolff Institute, Berlin, Germany

3 Charité – Universitätsmedizin Berlin, corporate member of Freie Universität Berlin and Humboldt-Universität zu Berlin, Institute of Biometry and Clinical Epidemiology, Berlin, Germany

4 German Federal Institute for Risk Assessment (BfR), German Centre for the Protection of Laboratory Animals (Bf3R), Berlin, Germany

5 Department of Trauma and Orthopedic Surgery, University Medical Center Hamburg-Eppendorf, Hamburg, Germany

6 Berlin Institute of Health at Charité – Universitätsmedizin Berlin, BIH Biomedical Innovation Academy, BIH Charité Clinician Scientist Program, Berlin, Germany

7 Department of Orthopaedic Surgery, Clinical Orthopaedic Surgery Hvidovre (CORH), Copenhagen University Hospital Hvidovre, Hvidovre, Denmark.

Correspondence: tazio.maleitzke@charite.de

Supplementary Figure S1. Nesting material. (a) Compact crinklets natural, (b) autoclaved compact crinklets natural, and (c) roll bedding (both SAFE®, Rosenberg, Germany) were provided as nest-building material. Nestlets were autoclaved before usage. Product images (a) and (c) are displayed with kind permission of J. Rettenmaier & Söhne GmbH + Co KG – SAFE®.

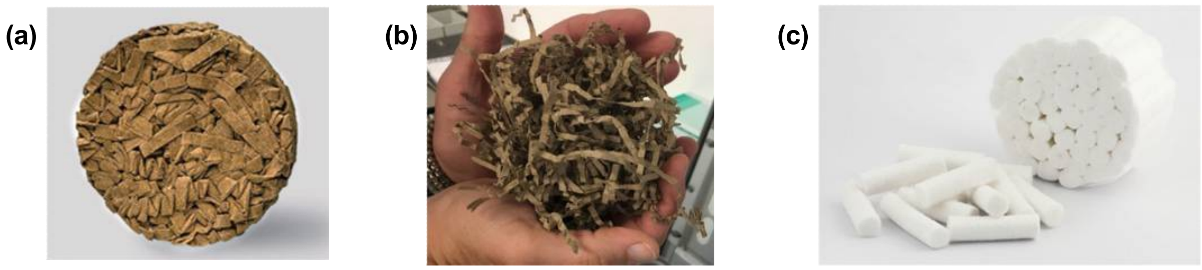

Supplementary Table S1. Mixed-effect models estimates (along with 95% CI) for differences between CAIA and CTRL animals, separately for inflammation (day 3 – 15), resolution (day 16 – 21), and repair and remodeling (day 22 – 48) phase.

**Parameter**		**Inflammation Day 3 – 15**	**Resolution Day 16 – 21**	**Repair and remodeling Day 22 – 48**
Nest score [unit]	Odds ratio[1]	1.02 (0.45, 2.32)	0.05 (0.00; 43.60)	0.72 (0.22; 2.34)
Nest score [unit]	Odds ratio[2]	0.82 (0.39; 1.72)	1.07 (0.32; 3.53)	0.96 (0.58; 1.57)
Body weight [g]	Regression coefficient[3]	-3.59 (-4.30, -2.87)	-2.50 (-3.45; -1.55)	-0.91 (-1.40; -0.42)
Grip strength [g]	Regression coefficient[3]	-22.63 (-27.67, -17.59)	-13.78 (-21.17; -6.40)	-6.04 (-9.80; -2.28)
Clinical arthritis score [unit]	Rate ratio[4]	17.35 (11.80, 25.50)	14.43 (5.24; 39.73)	6.89 (2.56; 18.50)
Ankle size [mm]	Regression coefficient[3]	0.13 (0.08; 0.18)	0.05 (-0.01; 0.12)	0.04 (0.02; 0.07)

[1] Derived from mixed logistic regression, using a nest score ≥ 3 as the outcome.

[2] Derived from mixed ordinal logistic regression.

[3] Derived from mixed linear regression.

[4] Derived from mixed negative binomial regression.

Supplementary Table S2. Repeated-measure Spearman correlation coefficients (along with 95% CI) between clinical parameters and nest scores for CAIA and CTRL mice divided by disease stages (Inflammation: day 3 – 15; Resolution: day 16 – 21; Repair and remodeling: day 22 – 48).

**Parameter**	**Group**	**Inflammation**<br>**Day 3 – 15**	**Resolution**<br>**Day 16 – 21**	**Repair and remodeling**<br>**Day 22 – 48**
Body weight [g]	all	0.14 (0.06; 0.22)	0.00 (-0.16; 0.16)	0.08 (0.01; 0.15)
	CAIA	0.22 (0.12; 0.31)	0.01 (-0.20; 0.22)	0.09 (-0.01; 0.19)
	CTRL	0.13 (0.00; 0.26)	-0.01 (-0.25; 0.23)	0.06 (-0.05; 0.17)
Grip strength [g]	all	0.15 (0.07; 0.23)	0.05 (-0.11; 0.20)	0.02 (-0.05; 0.09)
	CAIA	0.24 (0.14; 0.33)	0.15 (-0.05; 0.35)	-0.02 (-0.12; 0.08)
	CTRL	0.09 (-0.04; 0.22)	-0.03 (-0.27; 0.21)	0.07 (-0.04; 0.18)
Clinical arthritis score [unit]	all	0.10 (0.02; 0.18)	0.03 (-0.13; 0.18)	0.01 (-0.07; 0.08)
	CAIA	0.17 (0.07; 0.27)	0.07 (-0.13; 0.28)	-0.00 (-0.10; 0.10)
	CTRL	-0.08 (-0.21; 0.05)	-0.05 (-0.28; 0.20)	0.04 (-0.07; 0.15)
Ankle size [mm]	all	0.18 (0.10; 0.26)	0.15 (-0.00; 0.30)	0.02 (-0.05; 0.09)
	CAIA	0.19 (0.09; 0.28)	0.05 (-0.16; 0.25)	0.01 (-0.09; 0.11)
	CTRL	0.15 (0.03; 0.28)	0.27 (0.04; 0.48)	0.03 (-0.08; 0.14)

Supplementary Table S3. Sensitivity analyses using mixed ordinal logistic regression confirming associations between clinical parameters and nest score between day 3 and 15.

Parameter	Group	Mixed ordinal logistic regression	
		Unadjusted odds ratio (95% CI)	Adjusted odds ratio (95% CI)
Body weight [per 1 g]	all	1.21 (1.13; 1.30)	1.18 (1.09; 1.29)
	CAIA	1.28 (1.17; 1.40)	1.22 (1.10; 1.35)
	CTRL	1.16 (0.96; 1.40)	1.08 (0.88; 1.32)
Grip strength [per 10 g]	all	1.26 (1.16; 1.37)	1.31 (1.18; 1.45)
	CAIA	1.37 (1.22; 1.54)	1.48 (1.28; 1.71)
	CTRL	1.20 (1.02; 1.40)	1.18 (1.00; 1.38)
Clinical arthritis score [per 1 unit]	all	1.12 (1.06; 1.19)	1.24 (1.13; 1.36)
	CAIA	1.16 (1.09; 1.24)	1.33 (1.19; 1.49)
	CTRL	0.61 (0.36; 1.05)	0.53 (0.31; 0.92)
Ankle size [per 0.1 mm]	all	1.17 (1.08; 1.27)	1.04 (0.92; 1.17)
	CAIA	1.16 (1.06; 1.27)	0.98 (0.85; 1.13)
	CTRL	1.34 (1.03; 1.74)	1.28 (0.96; 1.71)
